# Can metabolic profiling provide a new description of osteoarthritis and enable a personalised medicine approach?

**DOI:** 10.1007/s10067-020-05106-3

**Published:** 2020-06-01

**Authors:** M. K. J. Jaggard, C. L. Boulangé, G. Graça, U. Vaghela, P. Akhbari, R. Bhattacharya, H. R. T. Williams, J. C. Lindon, C. M. Gupte

**Affiliations:** 1 Department of Orthopaedics & Trauma, Imperial College Healthcare NHS Trust, London, UK; 2 Department of Metabolism, Digestion and Reproduction, Imperial College London, London, UK; 3 Nestle Research Centre, Lausanne, Switzerland; 4 School of Medicine, Imperial College London, South Kensington, London, SW7 2AZ UK; 5 Department of Gastroenterology, Imperial College Healthcare NHS Trust, London, UK; 6 NIHR Imperial Biomedical Research Centre, Imperial College Healthcare NHS Trust, London, UK; 7 Department of Surgery and Cancer, Imperial College London, London, UK

**Keywords:** Metabolic profiling, Osteoarthritis, Personalised medicine, Metabonomics, Metabolomics

## Abstract

Osteoarthritis (OA) is a multifactorial disease contributing to significant disability and economic burden in Western populations. The aetiology of OA remains poorly understood, but is thought to involve genetic, mechanical and environmental factors. Currently, the diagnosis of OA relies predominantly on clinical assessment and plain radiographic changes long after the disease has been initiated. Recent advances suggest that there are changes in joint fluid metabolites that are associated with OA development. If this is the case, biochemical and metabolic biomarkers of OA could help determine prognosis, monitor disease progression and identify potential therapeutic targets. Moreover, for focussed management and personalised medicine, novel biomarkers could sub-stratify patients into OA phenotypes, differentiating metabolic OA from post-traumatic, age-related and genetic OA. To date, OA biomarkers have concentrated on cytokine action and protein signalling with some progress. However, these remain to be adopted into routine clinical practice. In this review, we outline the emerging metabolic links to OA pathogenesis and how an elucidation of the metabolic changes in this condition may provide future, more descriptive biomarkers to differentiate OA subtypes.

Metabolic profiling (*also known as* metabolomics, metabonomics and metabolic phenotyping) is emerging as a strategy to provide biomarker diagnostics capable of disease prediction, monitoring and early detection. It is defined as “the quantitative measurement of the dynamic multiparametric response of a living system to pathophysiological stimuli or genetic modification” [1]. Metabolites are the end points of molecular biology and variations in their profile reflect a response to the patient’s disease, environment, diet and lifestyle. Metabolic profiling has already established itself as a robust technique in oncology, epidemiology, gastrointestinal disease, metabolic syndrome (MeS), diabetes and cardiovascular disease [2–8]. Its scope for impact on clinical practice is exemplified by studies in which biomarker analysis can enhance the prediction of short term all-cause mortality, the profiling of chronic kidney disease and assessment of cardiovascular risk [9–11].

We can derive comprehensive descriptions of the thousands of metabolites present in a biofluid or tissue through complex analytical methods including but not exclusive to the following: nuclear magnetic resonance (NMR) spectrometry, liquid chromatography mass spectrometry (LC-MS), gas chromatography mass spectrometry (GC-MS) and capillary electrophoresis mass spectrometry (CE-MS) (Fig. 1)*.* The granular metabolic “fingerprint”, unique to specific disease states, can be compared with other disease subgroups or non-disease controls. Consequently, the technique can potentially delineate OA disease traits, symptoms, outcomes or treatment response. Analysis can be “untargeted”, where analysis does not target specific metabolites, or “targeted”, to focus on metabolite groups with associations of interests [1]. Nevertheless, interpreting metabolic data is challenging due to the significant number of variables and confounding factors. This is where computational multi-variate statistics can enable data interpretation and identify representative metabolites.

**Fig. 1:**
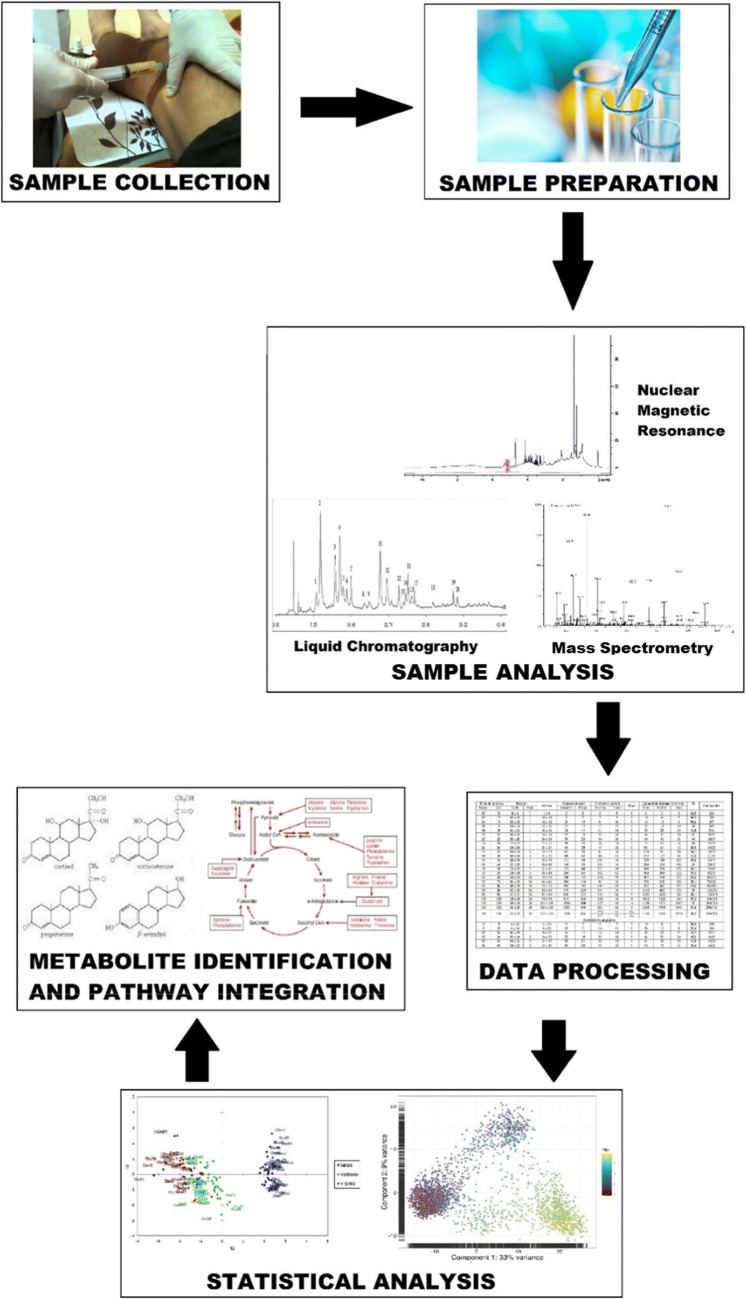
The step-wise process of metabolic profiling

## How does metabolic profiling differ from the other “big data” research methods?

The advantage of metabolic profiling is the “top down” representation of a disease phenotype, encompassing the genetics of disease in addition to environmental, lifestyle and dietary factors [12].

**Fig. 2:**
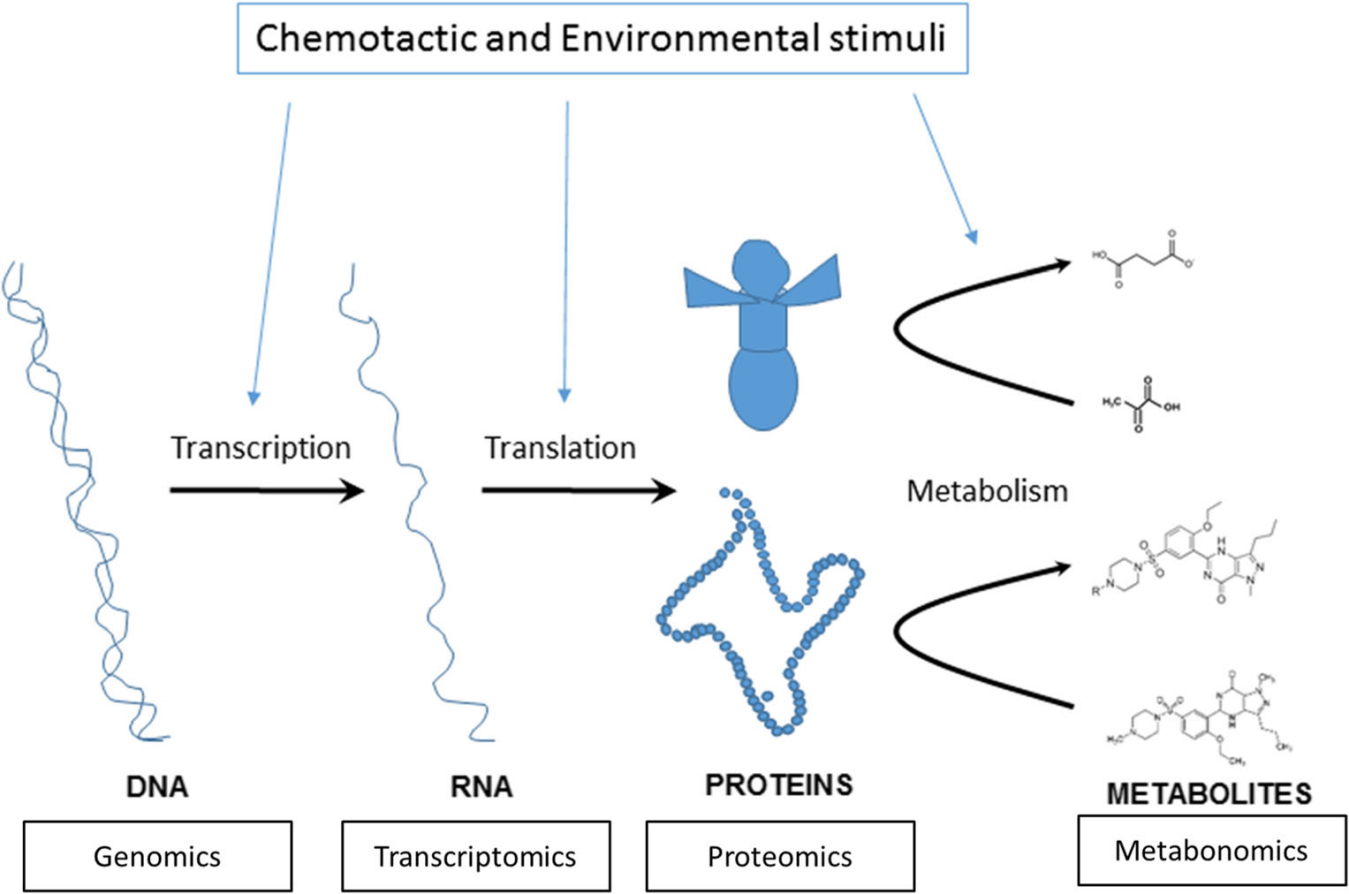
The methods of representing a biosystem using the “omic” techniques

Pathology in biological systems can be classified at the genome (genomic), transcriptome (transcriptomic), proteome (proteomic) and, ultimately, metabolome level (Fig. 2). Notably, these entities are not mutually exclusive, with cross-interactivity between the metabolome and other representations of the phenome [13]. Inter alia, metabolic profiling provides insights into the final stage of disease expression. The strength of metabolic data is in the patterns and relative abundance of small molecules.

Traditionally, clinicians have focused upon genomic and proteomic disease descriptions. However, pathogenesis or treatment response is modulated by both exogenous and host factors: immunosuppression, exercise, toxins, radiation, concurrent disease, diet and gut microbiome. Under circumstances where these factors have a substantial role in disease evolution, metabolic profiling, and its ability to account for this, provides a more attractive disease description.

## Search methods

In order to understand and research the metabolic influences of OA, the following search of the EMBASE and MEDLINE databases was performed:

1. “osteoarthritis” AND (“metabolic” OR “metabonomic” OR “metabolomic” OR “metabolism”)
2. (“synovial fluid” OR “cartilage” OR “synovium” OR “serum” OR “plasma” OR “urine”) AND (“NMR” or “Mass Spectrometry”)

A total of 8851 abstracts were identified and 28 reports contained suitable metabolic data.

## What is metabolic syndrome and how does it link to osteoarthritis?

MeS has a plethora of definitions and is associated with an increased risk of cardiovascular disease. Common to all definitions are insulin resistance, visceral obesity, atherogenic dyslipidaemia and hypertension [13]. In instituting the term “metabolic OA”, the link with metabolic syndrome has been pursued due to the shared mechanism of inflammation, oxidative stress, common metabolites and endothelial dysfunction in their aetiologies [14]. Therefore, metabolic syndrome underpins the hypotheses of OA metabolic description, having a large impact upon study design and execution of many studies.

A twofold increase in the risk of developing MeS has been demonstrated in patients suffering from OA [15]. Cardiovascular disease risk is elevated in female OA patients [16, 17]. Diabetes occurs more commonly in patients with radiological but not necessarily symptomatic osteoarthritis and confers a greater risk of developing bilateral hip osteoarthritis [18]. Inflammatory markers and pain scores are elevated in those suffering with OA and non-insulin-dependent diabetes mellitus compared with OA alone [19, 20]. A study of the serum of patients with OA showed an increase in total cholesterol compared with suitably matched controls [21]. This wide body of evidence for phenotypic variations and potentially more progressive osteoarthritis in the presence of metabolic disturbance has now resulted in OA being incorporated into some definition of MeS [22]. Nevertheless, what remains unclear from these predominantly retrospective studies is whether metabolic disturbances are a cause or effect of a proinflammatory state.

Previous studies have suggested that treating these metabolic disturbances, through dietary modification or direct-acting drug therapies, could slow or even halt the progression of OA [23]. Statin therapy in animal models showed promising reductions in catabolic OA cytokines and attenuated histological OA progression. Moreover, a 5-year longitudinal cohort study identified that atorvastatin significantly lowered the risk of developing knee pain [24]. The mechanism by which statins exert these effects remains unclear; however, reductions in IL-1, IL-6 and IL-8 and upregulation of nitric oxide synthase (NOS) have been postulated as potential mechanisms. These factors are intrinsically involved in the inflammatory pathogenesis of metabolic OA.

## Why is metabolism important in osteoarthritis?

OA is a multifactorial disease, which has a widely accepted metabolic variant. How and which key metabolic components establish and/or drive OA is not clear [14, 23, 25]. Broadly, it is perceived that the overexpression of proinflammatory mediators in OA is triggered through components of the MeS, including, for example, adipokines and advanced glycation end-products, from dyslipidaemic and hyperglycaemic states respectively.

Cohort studies suggest phenotypic variations in OA manifest as differing responses of pain and OA progression, BMI, sex and depression—all of which have been shown to differ metabolically [26]. Although these factors influence systemic phospholipid metabolism, a causal effect upon OA pathogenesis is yet to be established [27, 28]. By elucidating the role of metabolic stress in OA, we will gain insights into novel molecular targets for disease-modifying treatments.

## Which tissue types to sample and why?

The goal of metabolic analysis is to identify metabolic differences occurring secondary to disease or an intervention. Thus, in the context of disease analysis, the selection of body fluid or tissue requires careful consideration.

Typical biofluids such as urine and blood are favoured as advantageous due to a ubiquitous route of excretion, ease of sampling and mirroring any future clinical measurement of the disease state. A key shortcoming is their heterogeneity with many body systems resulting in spurious effects upon metabolite levels. Furthermore, in the context of OA, the relative effect of local joint disease towards systemic metabolism may be limited.

Local sampling of an affected tissue or fluid may prove more representative of the altered metabolic state and subsequently better describe disease aetiology or mechanism. However, sampling these fluids or tissues may be technically and ethically challenging [8].

In OA, sampling the synovial fluid or tissue can enable the investigation of specific changes in cartilage metabolism, where disease is most fulminant and major structural changes occur. Additionally, synovial fluid is solely responsible for supplying nutrients and removing waste products from the cartilage, thereby making it an attractive reservoir for the passage of OA-specific small molecules.

Alternatively, the synovium and intraarticular fat, as an adipokine source, may provide more insights into pro-inflammatory mediators of OA. Diseased cartilage may be studied directly to understand the small molecule changes associated with cartilage depolymerisation—nevertheless, clinical applicability will be limited due to the destructive nature of joint surface sampling.

## The metabolic discoveries of osteoarthritis

There are several archetypal metabolic profiles associated with OA. Synovial tissue culture from patients with varying degrees of osteoarthritis showed depletion in the branch chain amino acids (BCAA), amino acids (AA) and tricarboxylic acid (TCA) cycle intermediates with the predominance of lactate and pyruvate production [29]. This is consistent with the notion that the energetic stresses of OA mediate a shift to an anaerobic state [30–32]. It is worth noting, however, that this metabolic state is not specific to OA [33, 34]. Thus, clarification of the timing or degree of this metabolic disturbance compared with other disease states is necessary for OA-specific inferences. A more promising biomarker is plasma histidine, which is less concentrated in OA patients and as a singular measure may have value as a point-of-care diagnostic tool [34, 35].

The effect of osteoarthritic disease upon intra-articular proteoglycan destruction has been demonstrated in cartilage and synovial fluid [27, 30, 36, 37]. Whether this change is quantifiable as a proxy measure of the magnitude of joint destruction is unclear and requires larger, well-designed studies with more accurate descriptions of the intra-articular state. In principle, the measurement of biological monomers generated by the depolymerisation of structural molecules should reflect joint destruction, albeit, perhaps, with a non-linear relationship.

Lipid metabolism is an obvious target for metabolic study, due to its pro-inflammatory properties. OA synovial fluid has a marked increase in longer fatty acid chains [38]. Furthermore, with concurrent elevations in ketone bodies, this indicates a switch to fatty acid metabolism [30, 39, 40]. The results of the Canadian metabolome project indicate arginine depletion in plasma alongside an alteration to lipid species in OA [41]. Furthermore, the alterations in the lipid profile were more pronounced in the presence of diabetes—suggesting it could be a modality to isolate patients with metabolic OA [27]. A description of osteoarthritis populations revealed a separation of patients into lipid-specific classes, distinguished by virtue of glycophospholipid and sphingomyelin levels [42]. These lipid classes varied with age, obesity and response to pain, factors which confound with ageing-related and mechanical OA, but could still be linked to metabolic OA.

Mesenchymal stromal cell (MSC) differentiation towards cartilage anabolic cell lines has been shown to be affected in OA. These cell lines have been shown to favour glycolysis and uronic acid metabolism as disease progresses, specifically pentose and glucuronic acid [43].

The majority of studies to date have examined the joint as a biosystem in entirety. However, one must consider that mitochondrial DNA variants may drive metabolism and osteoarthritis disease. In addition to a disruption in oxidative phosphorylation is the effect upon macromolecule synthesis and maintenance. Whether the presence of the mitochondrial DNA variants is solely responsible for OA disease, metabolic associations and macromolecule catabolism is not clear [44]. However, despite the generation of reactive oxygen species, we do not believe this mechanism fully accounts for the inflammatory component of the disease or the altered lipid metabolism.

## What are the potential uses and outcomes of the metabolic data?

Descriptive reporting of the metabolic OA phenotype has identified associated metabolites. Nevertheless, to date, variations within OA subtypes have not been demonstrated or been able to explain the OA cohort variations in, for example, depression, pain and disease severity. While this can be attributed in the most part to inadequately powered studies, a metabolic description of the OA subtypes is required to determine potential associations with these clinically observed phenotypic variations.

**Fig. 3:**
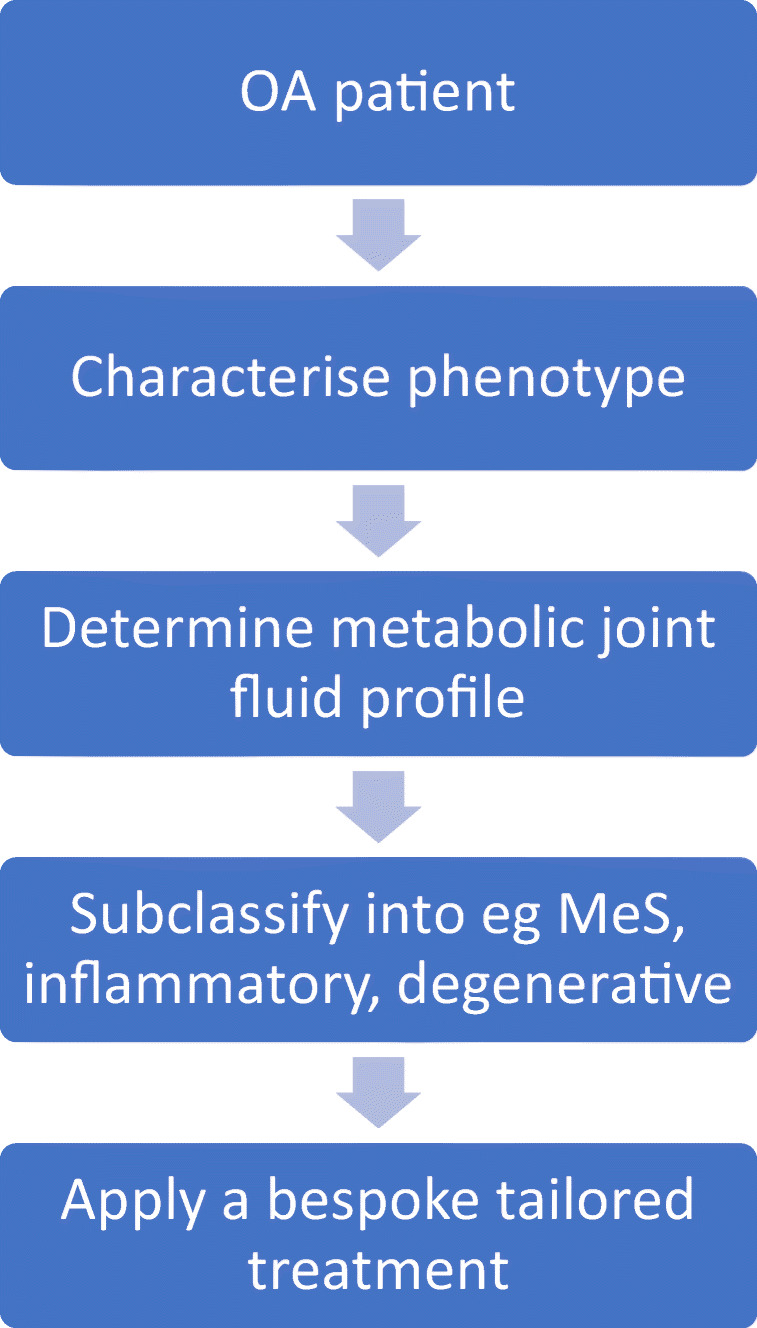
The osteoarthritis diagnostics and therapeutic workflow enabled by joint fluid metabolic profiling

If the aforementioned limitations are resolved, by characterising patients’ metabolic profiles, there is the potential to identify specific OA subtypes (Fig. 3). This model of care, with metabolic profiling as a diagnostic tool, can facilitate a paradigm shift from the present “one size fits all” OA management approach, which is indifferent to trauma-related or metabolic-induced OA. Metabolic profiling of joint fluid may provide a personalised insight into subsets of metabolic OA. Overall, this bespoke approach to treatment can only benefit patients, by permitting more efficacious, cost-effective treatment allocation and even the ability to predict treatment response [12, 45]. The latter can be achieved with the ability of metabolic data to provide advanced disease descriptions and a window into the real-time effects and interaction between treatment, lifestyle and dietary changes.

## Limitations of metabolic phenotyping and the over-reporting of results

As metabolic profiling is all-encompassing, confounding factors, including lifestyle, diet and exercise, pose the greatest limitation upon meaningful metabolic profile interpretation. This is further complicated by modern polypharmacy, which introduces additional metabolites and metabolism-altering drugs. These obstacles can be controlled by study design, sample numbers and sound statistical principles—no researcher should be naïve about their presence or influence.

Detection of metabolites is limited by metabolite similarity and instrument sensitivity. Instrument sensitivity continues to improve. However, a single technique will always have selective limitations and thus, using multiple analytical methods (NMR, LC-MS, GC-MS, CE-MS) is desirable.

A majority of multi-variate statistical models use linear regression and thus, any variable which has a non-linear association may be misrepresented—something frequently overlooked by investigators. These statistical models while powerful contribute to the exaggerated reporting of statistical significance. For example, one can easily use partial least squares discriminant analysis (PLS-DA) models to generate plots showing good pictorial separation of the sample classes; however, care must be taken that models are not overfitted and are appropriately validated. Hence, if the quality of model is not assured by using test sets or cross-validation, data can be misrepresented and generate false positives. Unfortunately, in some clinical studies, these errors have been carried through into publication.

## “Personalised medicine” and the impact of metabolic profiling

“Personalised medicine” is defined by NHS England as “a move away from a ‘one size fits all’ approach to the treatment and care of patients with a particular condition, to one which uses new approaches to better manage patients’ health and targets therapies to achieve the best outcomes in the management of a patient’s disease or predisposition to disease.” [44].

This is a concept widely held to be a viable product of metabolic profiling techniques and is being realised in the evaluation of atherosclerosis and the risk of developing cardiovascular disease [46]. The newfound interest and role of the metabolome in osteoarthritis make this goal achievable via a more detailed description of OA. This has potential for a more detailed and sub-categorised OA diagnosis with aetiology and treatments to match, possibly with metabolic targets. The importance of MSC subtypes and sub-cellular mitochondrial metabolism is not established but these metabolic perturbations are likely to be attractive targets for future study.

A great limitation to the success of these techniques in the research of OA is the lack of population-based studies. In order to address this, phenome centres have been created with significant financial and academic commitment, allowing centralisation of expertise and resources. There are now facilities capable of providing phenome data to rigorous standards for population-based studies. The geographical locations of these phenome centres lend themselves to make population comparisons. Furthermore, the possibility for metadata comparison and collation is much greater, due to agreed standards for experimental methods. To date, osteoarthritis research has not exploited these resources.

The power of “big data” tools like genomics and metabolic profiling comes through combined analysis, which effectively multiplies the descriptive power of one’s available data. Genome-wide associated studies (GWAS) have provided some OA genetic targets, primarily involved in gene regulation. Links have been demonstrated to genes involved in cartilage turnover (cartilage oligomeric matrix protein, COMP) and inflammation (transcription growth factor alpha, TGFA) [47, 48]. Marrying these findings to their downstream metabolic effects can allow a greater understanding of OA subtype aetiologies and widen the scope for therapeutic targets. Presently, in OA research, merged genotyping and advanced phenotyping are lacking.

Data analysis in using “big data” methods is challenging. The biggest limitation is often the data itself, due to a failure of collection or addressing the clinical question. Multi-variate linear regression models have become the standard analysis methods in interpreting metabolic profiling. Novel computational and machine learning algorithms have become more powerful and attracted sizeable investment. Limited metabolic profiling reports have applied these methods and one would expect an expansion going forward, with uncertain impact but hopeful advancement.

One of the barriers to clinical applications of metabolic phenotyping is its requirement for extensive and specialist analytical methods and equipment. The engineering of solutions capable of providing a complex, albeit, specific pattern of metabolite composition is evolving at pace. Handheld engineering solutions are now capable of sampling organic metabolites. Microchip technology allows multiple metabolite analysis and a coded result with the potential for robust, affordable and non-invasive testing [49]. In OA, these technologies could enable cost-effective early detection in asymptomatic individuals and sub-stratification in symptomatic individuals. Hence, we can envisage a future where a simple handheld breath or urine test can characterise disease and permit personalised treatment.

## Summary

The benefits of using the metabolic profile to provide an advanced description of osteoarthritis are numerous in both research and clinical practice. A detailed metabolic description of the OA patient can enable targeted therapy, ultimately reduce costs of treatment and allow predictions of one’s treatment journey. Drug development and treatment can be furthered by a greater understanding of an individual’s response to disease and therapy.

It is still not possible to accurately and reliably sub-stratify patients into OA phenotypes and identify metabolic OA. Nevertheless, robust studies are now being undertaken to inform our knowledge of OA subtypes and to suggest associated metabolites. Progress will require a population-based study with comprehensive clinical measures and in-depth metabolic measurements, with a view to describing the observed disease cohorts that are seen. Phenome centres will be pivotal in providing access to population data and expertise. This will move us away from a “one size fits all” model for OA treatment and deliver the promise of metabolic profiling.
